# Usefulness of the Córdoba Equation for Estimating Body Fat When Determining the Level of Risk of Developing Diabetes Type 2 or Prediabetes

**DOI:** 10.3390/medicina61040613

**Published:** 2025-03-27

**Authors:** Marta Marina Arroyo, Ignacio Ramírez Gallegos, Hernán Paublini, Ángel Arturo López-González, Pedro J. Tárraga López, Cristina Martorell Sánchez, Tomás Sastre-Alzamora, José Ignacio Ramírez-Manent

**Affiliations:** 1 Research ADEMA SALUD Group, University Institute for Research in Health Sciences (IUNICS), 07010 Palma, Spain; marinaarroyomarta@gmail.com (M.M.A.); ignacioramirezgallegos@gmail.com (I.R.G.); h.paublini@eua.edu.es (H.P.); c.martorell@eua.edu.es (C.M.S.); tsastre04@sonrie.com (T.S.-A.); joseignacio.ramirez@ibsalut.es (J.I.R.-M.); 2 Faculty of Dentistry, ADEMA University School, 07010 Palma, Spain; 3 Institut d’Investigació Sanitària de les Illes Balears (IDISBA), Health Research Institute of the Balearic Islands, 07004 Palma, Spain; 4 Health Service of the Balearic Islands, 07010 Palma, Spain; 5 Faculty of Medicine, University of Castilla la Mancha, 02071 Albacete, Spain; pjtarraga@sescam.jccm.es; 6 Faculty of Medicine, University of the Balearic Islands, 07122 Palma, Spain

**Keywords:** diabetes mellitus, prediabetes, visceral fat, body fat, body composition, ECORE-BF

## Abstract

*Background and Objectives*: Type 2 diabetes (T2D) and prediabetes represent major global health concerns, with obesity being a key risk factor. However, recent evidence suggests that the adipose tissue composition and distribution play a more critical role in metabolic dysfunction than the total body weight or body mass index (BMI). This study evaluates the predictive capacity of the Córdoba Equation for Estimating Body Fat (ECORE-BF) for identifying individuals at high risk of developing T2D and prediabetes. *Materials and Methods*: A cross-sectional study was carried out involving 418,343 Spanish workers. Body fat percentage was estimated using the ECORE-BF equation, and diabetes risk was assessed using validated predictive models, including the Finnish Diabetes Risk Score (FINDRISC), QDiabetes score (QD-score), and others. The discriminatory power of ECORE-BF in predicting T2D and prediabetes was assessed using receiver operating characteristic (ROC) curve analysis. *Results:* ECORE-BF showed a strong correlation with high-risk classifications across all diabetes risk scales. The area under the ROC curve (AUC) exceeded 0.95 for both men and women, demonstrating high predictive accuracy. *Conclusions:* Adipose tissue distribution, particularly visceral adiposity, is a central factor in metabolic dysfunction. ECORE-BF provides a cost-effective alternative for large-scale T2D and prediabetes risk assessment. Future research should explore the impact of visceral adipose tissue reduction on diabetes prevention and the integration of estimation scales into clinical and public health strategies.

## 1. Introduction

Diabetes mellitus is among the most common chronic non-communicable diseases, affecting millions of individuals worldwide [1]. Its increasing prevalence and significant impact on patients’ quality of life have made it a critical public health concern [2]. Type 2 diabetes (T2D), responsible for approximately 90–95% of all diabetes cases worldwide, arises from a combination of insulin resistance and impaired insulin secretion by pancreatic beta cells [3,4,5]. The progression of the disease is influenced by multiple risk factors, including overweight, obesity [6], physical inactivity [7], and genetic predisposition [8]. Early detection [9] and proper management are essential to prevent long-term complications [10] and improve patient outcomes [11].

Type 2 diabetes has increased globally [12] and is one of the leading causes of morbidity and mortality [13]. In 2021, 537 million adults had diabetes, with projections exceeding 700 million by 2045 [14] if effective prevention and control strategies are not implemented [15]. It is more prevalent in low- and middle-income countries, where urbanization, lifestyle changes, and limited healthcare access contribute to its rise [16].

Sociodemographic factors influence the distribution of diabetes [17]. Ethnic groups such as Hispanics, African Americans, U.S. Indigenous populations [18], South Asians [19], and populations from the Middle East [20] and Africa [21] are at higher risk. Additionally, population aging increases the incidence of type 2 diabetes due to age-related insulin resistance [22].

Diabetes-related complications are broadly classified into microvascular and macrovascular complications. Microvascular complications of diabetes include retinopathy, nephropathy, and neuropathy. Retinopathy can lead to irreversible blindness [23], nephropathy is a major cause of chronic kidney disease and dialysis [24], and neuropathy increases the risk of foot ulcers and lower limb amputations due to sensory loss [25]. Macrovascular complications include cardiovascular diseases such as ischemic heart disease [26], cerebrovascular disease [27], and peripheral artery disease [28]. Hyperglycemia, dyslipidemia, and hypertension contribute to atherosclerosis [29], making cardiovascular diseases the leading cause of mortality in type 2 diabetes [30].

Early identification of individuals at high risk of developing T2D is crucial for prevention and disease management [31]. Several validated risk assessment tools have been developed to estimate the likelihood of diabetes onset in specific populations. The most widely used scales include the following:

(1) Finnish Diabetes Risk Score (FINDRISC) [32];
(2) American Diabetes Association (ADA) Diabetes Risk Test [33];
(3) QDiabetes score [34].

These tools assess an individual’s risk by incorporating variables such as age, body mass index (BMI), family history of diabetes, physical activity, diet, and blood pressure.

The implementation of these risk assessment tools enables the early detection of individuals in a prediabetic state [35], facilitating timely intervention strategies such as lifestyle modifications and medical follow-up. Moreover, these tools play a crucial role in shaping public health policies designed to reduce the societal burden of diabetes [36,37].

Obesity is a primary risk factor for T2D and represents a growing global public health challenge [38]. The excessive accumulation of adipose tissue, particularly in the abdominal region, is strongly linked to insulin resistance, a key pathophysiological mechanism underlying the development of T2D [39]. Visceral obesity promotes a pro-inflammatory and oxidative stress state, marked by the release of inflammatory cytokines such as tumor necrosis factor-alpha (TNF-α) [40] and interleukin-6 (IL-6) [41], which impair insulin action in peripheral tissues.

Moreover, dysfunctional adipose tissue in obese individuals secretes lower levels of adiponectin, a hormone that enhances insulin sensitivity and regulates lipid and glucose metabolism. Reduced adiponectin levels, coupled with increased insulin resistance, promote chronic hyperglycemia and the progression toward T2D [42]. Additionally, obesity is associated with beta-cell dysfunction, compromising the body’s ability to maintain glycemic homeostasis [43].

Epidemiological studies have demonstrated that weight reduction through lifestyle interventions, including dietary modifications and increased physical activity, can delay or prevent the onset of T2D in individuals with obesity and prediabetes [44]. In this regard, the Diabetes Prevention Program (DPP) has shown that modest weight loss (5–10% of initial body weight) can significantly reduce the risk of developing T2D [45]. Consequently, obesity prevention and treatment are critical strategies for addressing the global diabetes epidemic.

Type 2 diabetes poses a significant epidemiological burden and is associated with multiple complications. The use of validated risk assessment tools and the implementation of preventive strategies are essential to mitigate its impact. Given the strong link between obesity and T2D, it is imperative to develop effective public health policies that promote healthy lifestyles and reduce the incidence of the disease.

The objective of this study is to evaluate the utility of the Córdoba Equation for Estimating Body Fat (ECORE-BF) as a predictor of elevated values in various risk assessment scales for T2D and prediabetes.

## 2. Materials and Methods

This study is a descriptive, cross-sectional analysis conducted between January 2017 and December 2019, involving a total of 418,343 workers in Spain (246,061 men and 172,282 women) from various regions. The participants were selected from employees undergoing routine occupational health examinations in the companies that agreed to take part in this study.

To qualify for inclusion, individuals needed to be between 18 and 69 years old, employed under a contract with one of the participating companies, and not on temporary leave due to health-related reasons at the time of assessment, and they must have provided informed consent to participate in this research, allowing the collected data to be used for epidemiological analysis.

**Figure 1:**
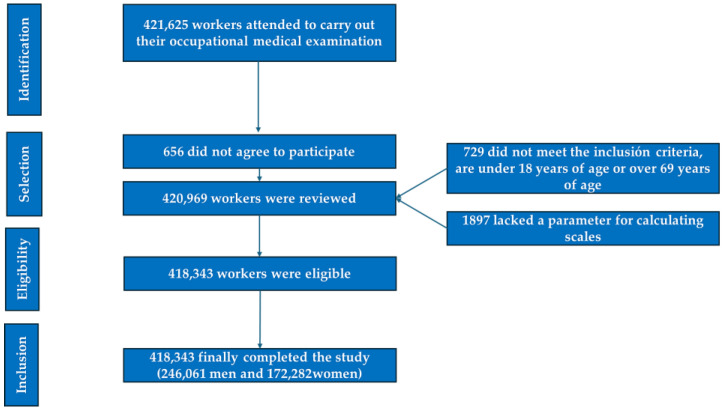
Flowchart for selecting employees in this study.

Figure 1 presents a flowchart illustrating the selection process of the employees included in this study.

### 2.1. Measurement and Data Collection

Prior to data collection, this study’s medical and nursing personnel standardized the measurement protocols. These healthcare professionals were responsible for conducting clinical, analytical, and anthropometric assessments, including waist circumference, weight, and height.

Height and weight measurements were obtained using a SECA 700 model weighing scale. Waist circumference was measured with SECA measuring tape while the participant stood upright, with the lower extremities together, the trunk erect, and the abdomen relaxed. The tape was positioned parallel to the floor at the level of the last floating rib to ensure accuracy.

Blood pressure readings were taken while the participant was seated, following a minimum rest period of 10 min, using a calibrated automatic sphygmomanometer (OMRON M3). Three consecutive measurements were recorded at 60 s intervals, and the mean value was calculated.

Biochemical parameters were assessed after a fasting period of at least 12 h. Triglyceride, glucose, and total cholesterol levels were determined using automated enzymatic methods. High-density lipoprotein (HDL) cholesterol levels were measured with dextran sulfate-MgCl_2_ precipitation techniques. Low-density lipoprotein (LDL) cholesterol was indirectly calculated using the Friedewald equation [46].

All biochemical values were reported in mg/dL. Glycemic status was classified based on the criteria established by the American Diabetes Association (ADA): fasting glucose levels below 100 mg/dL were considered normal, while values between 100 and 125 mg/dL indicated prediabetes or impaired fasting glucose, and readings exceeding 125 mg/dL were indicative of diabetes [47].

### 2.2. Body Fat Estimation and Risk Scales

Body fat percentage was estimated using the Córdoba Equation for Estimating Body Fat (ECORE-BF), calculated as follows:

ECORE-BF = 97.102 + 0.123 (age) + 11.9 (sex) + 35.959 (lnBMI) [48].

Age = in years old at that time.

Sex = 0 for men and 1 for women.

lnBMI = natural logarithm of the body mass index.

Cut-off points for obesity = men > 25%, women > 35%.

A previous study conducted by our research group demonstrated a high concordance (0.998) between ECORE-BF and the Clínica Universitaria de Navarra Body Adiposity Estimator (CUN-BAE), which is considered the gold standard for body fat estimation, as validated in a study published in *Diabetes Care* [49].

The risk of developing type 2 diabetes was evaluated using multiple validated predictive models:

(1) FINDRISC: This index takes into account sex, age, BMI, waist circumference, physical activity, consumption of fruits and vegetables, use of antihypertensive medication, history of hyperglycemia, and family history of diabetes. A score higher than 15 indicates a high risk [50].
(2) QDiabetes score (QD-score): This model incorporates variables including age, sex, ethnicity, height, weight, blood glucose levels, smoking status, history of stroke, family history of diabetes, use of antihypertensive medication, presence of depression or schizophrenia, and use of steroids or statins. It also considers a history of polycystic ovary syndrome or gestational diabetes. In the absence of established cut-off points, a relative risk of ≥3 was defined as indicative of a high-risk profile [51].
(3) Canrisk: This index includes information on sex, age, physical activity, fruit and vegetable consumption, history of hypertension, past hyperglycemia, family history of diabetes, ethnicity, and education level. A score above 43 suggests a higher diabetes risk [52].
(4) Trinidad Risk Assessment Questionnaire for Type 2 Diabetes Mellitus (TRAQ-D): This model accounts for age, sex, BMI, smoking history, family history of diabetes, and ethnicity [53].
(5) Prediabetes risk scale for Qatar (PRISQ Scale): This tool assesses prediabetes risk, incorporating factors such as age, sex, waist circumference, BMI, and blood pressure. In the Qatari population, a score ≥16 indicates a high risk, a threshold that was also found to be valid in the Spanish population [54].

With respect to the smoking status, individuals were classified as smokers if they had smoked at least one cigarette per day (or an equivalent tobacco intake in other forms) or had quit smoking within the last year.

The classification of workers by socioeconomic status was based on the 2011 Spanish National Occupational Classification (CNO-11) and the criteria established by the Spanish Society of Epidemiology’s Social Determinant Groups: Class I (directors/managers, university professionals, athletes, and artists), Class II (intermediate occupations and self-employed workers without employees), and Class III (unskilled workers) [55].

### 2.3. Statistical Analysis

A descriptive statistical analysis was performed by calculating the frequency and distribution of categorical variables. Since the quantitative variables followed a normal distribution, means and standard deviations (SDs) were computed. For comparisons between independent groups, Student’s *t*-test was applied to continuous variables, while the Chi-square test was used for categorical data. Fisher’s exact test was conducted when necessary. Receiver operating characteristic (ROC) curve analysis was utilized to assess the discriminatory power of ECORE-BF in predicting type 2 diabetes and prediabetes. The optimal cut-off values were determined mathematically from the ROC curves. All statistical analyses were conducted using IBM SPSS Statistics v29.0 for Windows, with a significance level set at *p* < 0.05.

## 3. Results

Table 1 presents the clinical and demographic characteristics of the study population. In general, men exhibited higher or less favorable values for most anthropometric, clinical, and biochemical parameters.

The predominant age group among participants was 30–49 years, and the majority of workers belonged to social class III according to the established classification. Approximately one-third of the participants were identified as current smokers.

Table 2 and Table 3 present the mean values and prevalence of obesity according to the ECORE-BF scale, stratified by the risk levels in diabetes and prediabetes assessment scales. In both cases, individuals classified as high risk for diabetes and prediabetes exhibit higher values compared to their lower-risk counterparts. The observed differences are consistently statistically significant (*p* < 0.001).

**Figure 2:**
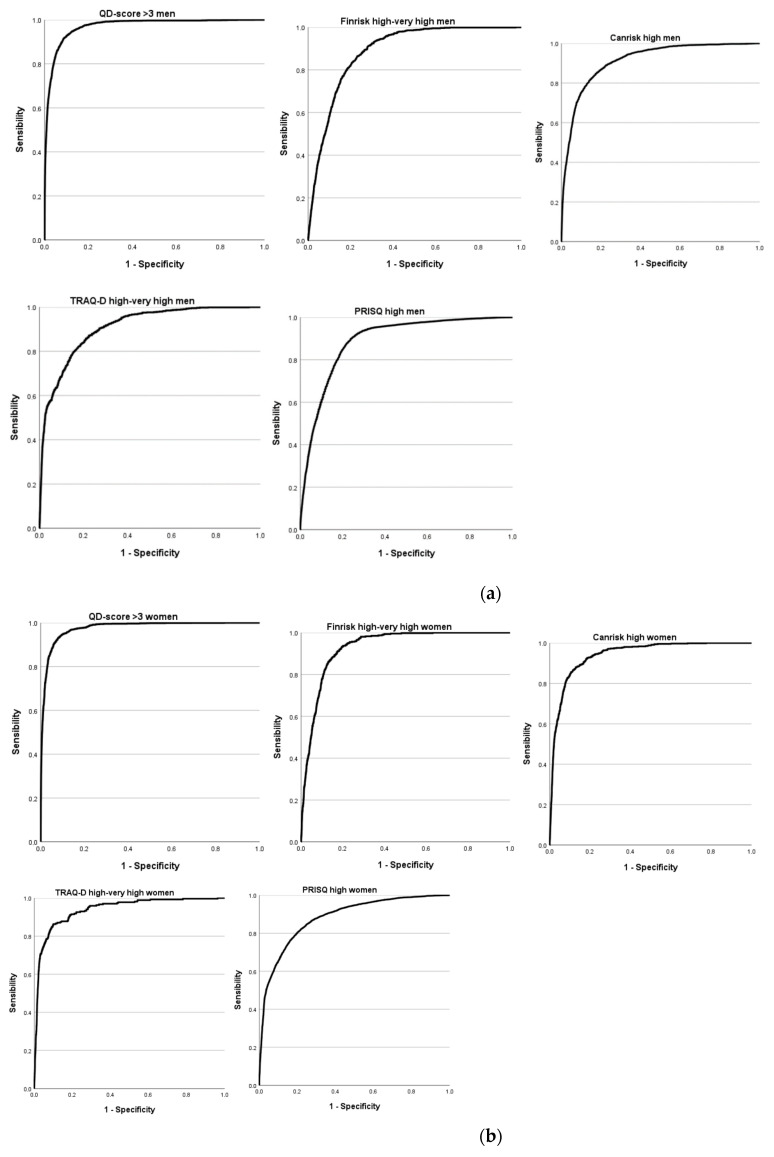
(**a**). ROC curves to estimate the value of ECORE-BF for predicting a high risk of prediabetes and diabetes in men. (**b**). ROC curves to estimate the value of ECORE-BF for predicting a high risk of prediabetes and diabetes in women.

As shown in Figure 2a,b and Table 4, the ECORE-BF scale serves as a strong predictor of high values in diabetes and prediabetes risk scales, as indicated by the areas under the curve (AUCs). The AUC values are particularly high for a QD-score > 3, specifically 0.970 (95% CI: 0.969–0.971) for men and 0.976 (95% CI: 0.975–0.977) for women. Additionally, the AUC values remain high across all other diabetes and prediabetes risk scales. In the diabetes risk scales, the AUC values are higher in women, whereas for prediabetes risk scales, they are slightly higher in men.

## 4. Discussion

In our study, the ECORE-BF scale has demonstrated a high predictive value for identifying elevated values in different T2D and prediabetes risk scales, particularly a high QD-score.

Obesity is a well-established risk factor for T2D [56] and prediabetes [57] due to its contribution to insulin resistance and pancreatic beta-cell dysfunction. However, the distribution and composition of adipose tissue, rather than mere excess weight, appear to play a crucial role in the development of metabolic disorders [58]. Although the body mass index (BMI) is widely used as an indicator of obesity, it does not distinguish between fat mass and lean mass, nor does it consider the regional distribution of adipose tissue [59].

In this context, the assessment of body fat, whether through objective techniques such as dual-energy X-ray absorptiometry (DXA) [60] or bioelectrical impedance analysis (BIA) [61]; magnetic resonance imaging (MRI) [62]; or validated estimation scales such as the ECORE-BF equation [63], plays a crucial role as it allows for a more precise characterization of obesity and its metabolic implications. This discussion explores the relationship between body fat, particularly visceral adiposity, and the risk of T2D and prediabetes, emphasizing findings obtained through both objective measurements and estimation scales. A concordance analysis was performed using Pearson’s correlation coefficient to evaluate the relationship between bioimpedance (BIA) and the ECORE-BF formula. The obtained result was a value of 0.915, indicating a high correlation and strong agreement between both methods for measuring body fat.

The location and function of adipose tissue significantly influence metabolic outcomes. Subcutaneous adipose tissue (SAT), predominantly found in the gluteofemoral region, has been shown to have protective effects against metabolic diseases [64]. In contrast, visceral adipose tissue (VAT), which accumulates in the abdominal cavity and surrounds vital organs, is strongly associated with insulin resistance [65], inflammation [66], and an increased risk of diabetes [67].

VAT contributes to metabolic dysfunction through several mechanisms:

1. Increased lipolysis and free fatty acid release: VAT is more metabolically active than SAT and releases higher amounts of free fatty acids (FFAs) into the portal circulation, contributing to hepatic insulin resistance [68].
2. Secretion of pro-inflammatory cytokines: Visceral adipose tissue (VAT) generates inflammatory cytokines, including tumor necrosis factor-alpha (TNF-α) and interleukin-6 (IL-6), which disrupt insulin signaling and contribute to a state of chronic low-grade inflammation [69].
3. Ectopic fat accumulation: Excess visceral adipose tissue (VAT) can result in lipid accumulation in non-adipose tissues, such as the liver, muscles, and pancreas, further aggravating insulin resistance and beta-cell dysfunction [70].

Studies using MRI and DXA have consistently shown that the VAT volume correlates more strongly with insulin resistance and T2D risk than the total fat mass or BMI [71]. These findings underscore the importance of assessing the body fat distribution rather than relying solely on BMI-based obesity classification.

There are different objective techniques for evaluating body fat and its association with diabetes risk:

1. Dual-Energy X-ray Absorptiometry (DXA). DXA is widely regarded as the gold standard for analyzing body composition, providing precise measurements of total fat mass, lean mass, and visceral fat [72]. Studies have shown that a higher VAT volume and lower lean mass, as measured by DXA, significantly predict T2D incidence, independent of BMI [73]. For example, a prospective cohort study involving more than 1800 adults found that individuals in the highest VAT tertile (measured by DXA) had a 3.5-fold higher risk of developing T2D compared to those in the lowest tertile [74].
2. Magnetic Resonance Imaging (MRI) and Computed Tomography (CT). Both MRI and CT provide highly accurate evaluations of adipose tissue distribution, enabling differentiation between VAT and SAT [75]. Some studies using MRI have demonstrated that progressive VAT accumulation is a key predictor of impaired insulin sensitivity and T2D development [76].

Given the high cost and limited accessibility of DXA, MRI, and CT, several validated estimation scales have been developed to assess body fat and its distribution. Among them, the ECORE-BF scale has been validated against reference methods and provides a reliable estimate of the body fat percentage [49].

Recent studies align with our findings and have demonstrated that the ECORE-BF scale exhibits a high predictive capacity for diabetes and prediabetes, with areas under the receiver operating characteristic (ROC) curve (AUC) exceeding 0.95 in both men and women [77].

The mean values and obesity prevalence, assessed using the ECORE-BF scale, were analyzed and stratified according to the risk levels in the diabetes and prediabetes scales. In both cases, it was observed that individuals classified as high-risk for developing diabetes or prediabetes had significantly higher obesity values compared to those classified in the lower-risk groups. These differences were consistent across both sexes and in the various risk categories, reflecting a strong relationship between obesity and metabolic risk. The results obtained were statistically significant, with a *p*-value < 0.001, highlighting the importance of obesity as a crucial risk factor in the prevention and diagnosis of diabetes and prediabetes. This finding underscores the utility of the ECORE-BF scale in identifying individuals at risk, facilitating early intervention.

Our results reveal that the ECORE-BF scale maintains a remarkable discriminatory ability across all the evaluated risk scales, not only for diabetes but also for prediabetes. Although the AUC values are high in both sexes, there is a trend in which women present higher AUC values in the diabetes risk scales, while in the prediabetes risk scales, the values are slightly higher in men. This pattern could reflect biological or metabolic differences between the sexes that affect the prevalence and progression of the disease.

These findings emphasize the relevance of the ECORE-BF scale as an assessment tool for the early identification of individuals at risk of developing diabetes or prediabetes, which could facilitate the implementation of personalized and effective prevention strategies, particularly in clinical and primary care settings.

The ECORE-BF scale was validated by comparing it with the CUN BAE (concordance index of 0.998 in over 196,000 people), demonstrating that it is as useful as this scale in the same populations, but its calculation is much simpler and requires far fewer parameters [78]. This scale has been validated in the Spanish population, suggesting its potential applicability in other European populations. However, since its formula includes lnBMI, we consider that its use in different ethnic groups and races requires adjustment of BMI cut-off points and prior validation in each specific population. In particular, the Asian population generally has a lower BMI but a higher body fat percentage compared to the Caucasian population. For this reason, the standard threshold for defining obesity (BMI ≥ 30.0 kg/m^2^) is inadequate, as it does not accurately reflect their body composition and associated metabolic risk. To ensure its correct application in these populations, it is essential to establish specific thresholds adjusted to their anthropometric and metabolic characteristics. Additionally, further studies should be conducted to validate the scale in other populations with different adiposity profiles and cardiovascular risk levels [79].

### Strengths and Limitations

One of the main strengths of this study is its large sample size, along with the comprehensive evaluation of type 2 diabetes and prediabetes risk, incorporating analyses from five different assessment scales.

Additionally, since the participants were drawn from nine autonomous Spanish communities with substantial populations, the findings may be considered representative of the Spanish population.

Among the main limitations, this study lacks direct body fat measurements, relying instead on estimates derived from a validated scale. Moreover, the cross-sectional design limits the ability to establish causal relationships, allowing only for the identification of associations.

Another limitation is the exclusion of unemployed individuals, retirees, and those under 18 or over 69 years old. However, given the large sample size, these exclusions are unlikely to significantly affect the validity of the results.

The ECORE-BF formula has been validated for the Spanish population, and to apply it to other ethnicities or races, appropriate adjustments must be made.

In the female population, depending on individuals’ hormonal statuses (pre- or postmenopausal), there are differences in how fat is deposited in the body and how the diabetes risk grows. Since we did not know the participants’ dates of menopause onset, a corresponding adjustment could not be made.

## 5. Conclusions

Although obesity is a major risk factor for the development of T2D and prediabetes, the adipose tissue composition and distribution are more determinant than the total body weight or BMI. Objective measurements such as DXA, MRI, and BIA have demonstrated that visceral adiposity is a central factor in metabolic dysfunction.

Additionally, body fat estimation scales such as ECORE-BF provide a cost-effective alternative for predicting the T2D and prediabetes risk in large populations.

Future research should focus on longitudinal studies analyzing the impact of VAT reduction on diabetes prevention, as well as the integration of estimation scales into clinical practice and public health strategies.

## Figures and Tables

**Table 1:** Characteristics of the population.

	Men	Women	Total	
	n = 246,061	n = 172,282	n = 418,343	
	Mean (SD)	Mean (SD)	Mean (SD)	*p*-Value
Age	40.6 (11.1)	39.6 (10.8)	40.2 (11.0)	<0.0001
Height	174.6 (7.0)	161.8 (6.5)	169.4 (9.3)	<0.0001
Weight	81.4 (14.7)	66.2 (14.0)	75.1 (16.2)	<0.0001
Waist	86.2 (11.1)	74.8 (10.6)	81.5 (12.2)	<0.0001
Systolic BP	128.2 (15.5)	117.4 (15.7)	123.7 (16.5)	<0.0001
Diastolic BP	77.8 (11.0)	72.6 (10.4)	75.6 (11.0)	<0.0001
Cholesterol	192.6 (38.9)	190.6 (35.8)	191.8 (37.7)	<0.0001
HDL-c	50.3 (8.5)	56.8 (8.7)	53.0 (9.1)	<0.0001
Non-HDL cholesterol	144.9 (41.4)	139.8 (39.6)	142.9 (40.8)	<0.0001
LDL-c	118.0 (36.7)	116.1 (34.8)	117.2 (35.9)	<0.0001
Triglycerides	123.7 (86.4)	89.1 (46.2)	109.5 (74.6)	<0.0001
Glycemia	93.3 (21.3)	87.8 (15.1)	91.0 (19.2)	<0.0001
	%	%	%	*p*-value
Under 30 years	18.8	20.7	19.6	<0.0001
30–39 years	27.6	29.7	28.4	
40–49 years	30.0	29.6	29.9	
50–59 years	19.7	16.8	18.5	
60–69 years	3.9	3.2	3.6	
Economic class I	4.9	6.9	5.7	<0.0001
Economic class II	14.9	23.4	18.4	
Economic class III	80.3	69.7	75.9	
No tobacco consumption	66.6	67.2	66.9	<0.0001
Tobacco consumption	33.4	32.8	33.2	

BP Blood pressure. HDL-c High-density lipoprotein-cholesterol. LDL-C Low-density lipoprotein-cholesterol.

**Table 2:** Mean ECORE-BF values based on different risk scale scores for type 2 diabetes and prediabetes, stratified by sex.

		Men			Women	
	n	Mean (SD)	*p*-Value	n	Mean (SD)	*p*-Value
QD-score < 3	224,002	25.0 (4.9)	<0.001	153,641	33.8 (5.6)	<0.001
QD-score > 3	22,059	35.9 (3.8)		18,641	47.6 (4.4)	
FINDRISC low-normal	237,936	25.2 (4.5)	<0.001	167,989	32.6 (5.1)	<0.001
FINDRISC high-very high	8125	34.6 (4.4)		4293	47.9 (5.2)	
Canrisk low-normal	257,763	25.3 (4.1)	<0.001	166,377	36.5 (5.4)	<0.001
Canrisk high	28,298	33.7 (4.6)		5905	48.3 (5.3)	
TRAQ-D low	238,776	25.6 (5.1)	<0.001	169,849	36.3 (6.0)	<0.001
TRAQ-D high-very high	7285	36.0 (5.2)		2433	49.6 (5.9)	
PRISQ normal	119,402	21.8 (4.6)	<0.001	129,519	32.6 (5.4)	<0.001
PRISQ high	126,659	29.2 (4.6)		42,763	42.2 (6.2)	

ECORE-BF Córdoba Equation for Estimating Body Fat. QD-score Q diabetes score. TRAQ-D Trinidad Risk Assessment Questionnaire for Type 2 Diabetes Mellitus. PRISQ Prediabetes risk in Qatar. SD Standard body fat.

**Table 3:** Prevalence of elevated ECORE-BF values based on risk levels for prediabetes or type 2 diabetes, categorized by different scales and stratified by sex.

		Men			Women	
ECORE-BF Obesity	n	%	*p*-Value	n	%	*p*-Value
QD-score < 3	224,002	27.6	<0.001	153,641	40.3	<0.001
QD-score > 3	22,059	99.3		18,641	99.8	
FINDRISC low-normal	237,936	34.4	<0.001	167,989	30.8	<0.001
FINDRISC high-very high	8125	99.7		4293	99.5	
Canrisk low-normal	257,763	47.0	<0.001	166,377	43.2	<0.001
Canrisk high	28,298	98.5		5905	99.7	
TRAQ-D low	238,776	51.4	<0.001	169,849	44.4	<0.001
TRAQ-D high-very high	7285	99.6		2433	99.8	
PRISQ normal	119,402	19.5	<0.001	129,519	30.9	<0.001
PRISQ high	126,659	84.1		42,763	88.2	

ECORE-BF Córdoba Equation for Estimating Body Fat. QD-score Q diabetes score. TRAQ-D Trinidad Risk Assessment Questionnaire for Type 2 Diabetes Mellitus. PRISQ Prediabetes risk in Qatar.

**Table 4:** Areas under the curve and ECORE-BF cut-off points, along with their sensitivity, specificity, and Youden index, for predicting high-risk values on different risk scales for prediabetes and type 2 diabetes, stratified by sex.

	Men n = 246,061		Women n = 172,282	
	AUC (95% CI)	Cut-off-Sens-Specif-Youden	AUC (95% CI)	Cut-off-Sens-Specif-Youden
QDScore > 3	0.970 (0.969–0.971)	31.5-91.4-91.3-0.827	0.976 (0.975–0.977)	42.0-92.4-92.3-0.847
FINDRISC high-very high	0.886 (0.884–0.889)	30.3-81.0-80.9-0.619	0.929 (0.926–0.932)	42.3-86.4-86.4-0.728
Canrisk high	0.912 (0.910–0.914)	29.6-83.7-83.5-0.672	0.941 (0.938–0.943)	42.3-87.7-87.1-0.758
TRAQ-D high-very high	0.907 (0.903–0.910)	30.5-82.3-82.0-0.643	0.938 (0.933–0.944)	42.9-87.1-87.1-0.742
PRISQ high	0.888 (0.886–0.889)	25.3-81.8-81.7-0.635	0.881 (0.879–0.883)	37.1-80.3-80.0-0.603

QDScore Q diabetes score. TRAQ-D Trinidad Risk Assessment Questionnaire for Type 2 Diabetes Mellitus. PRISQ Prediabetes risk in Qatar. AUC Area under the curve.

## Data Availability

The original contributions presented in this study are included in the article. Further inquiries can be directed to the corresponding author.

## References

[1] Arroyave F., Montaño D., Lizcano F. (2020). Diabetes Mellitus Is a Chronic Disease that Can Benefit from Therapy with Induced Pluripotent Stem Cells. Int. J. Mol. Sci..

[2] Kolarić V., Svirčević V., Bijuk R., Zupančič V. (2022). Chronic complications of diabetes and quality of life. Acta Clin. Croat..

[3] Eizirik D.L., Pasquali L., Cnop M. (2020). Pancreatic β-cells in type 1 and type 2 diabetes mellitus: Different pathways to failure. Nat. Rev. Endocrinol..

[4] Galicia-Garcia U., Benito-Vicente A., Jebari S., Larrea-Sebal A., Siddiqi H., Uribe K.B., Ostolaza H., Martín C. (2020). Pathophysiology of Type 2 Diabetes Mellitus. Int. J. Mol. Sci..

[5] Mizukami H., Kudoh K. (2022). Diversity of pathophysiology in type 2 diabetes shown by islet pathology. J. Diabetes Investig..

[6] Ruze R., Liu T., Zou X., Song J., Chen Y., Xu R., Yin X., Xu Q. (2023). Obesity and type 2 diabetes mellitus: Connections in epidemiology, pathogenesis, and treatments. Front. Endocrinol..

[7] Di Murro E., Di Giuseppe G., Soldovieri L., Moffa S., Improta I., Capece U., Nista E.C., Cinti F., Ciccarelli G., Brunetti M. (2023). Physical Activity and Type 2 Diabetes: In Search of a Personalized Approach to Improving β-Cell Function. Nutrients.

[8] Shojima N., Yamauchi T. (2023). Progress in genetics of type 2 diabetes and diabetic complications. J. Diabetes Investig..

[9] Zhang J., Zhang Z., Zhang K., Ge X., Sun R., Zhai X. (2023). Early detection of type 2 diabetes risk: Limitations of current diagnostic criteria. Front. Endocrinol..

[10] Majety P., Lozada Orquera F.A., Edem D., Hamdy O. (2023). Pharmacological approaches to the prevention of type 2 diabetes mellitus. Front. Endocrinol..

[11] Khan M.A.B., Hashim M.J., King J.K., Govender R.D., Mustafa H., Al Kaabi J. (2020). Epidemiology of Type 2 Diabetes-Global Burden of Disease and Forecasted Trends. J. Epidemiol. Glob. Health.

[12] GBD 2021 Diabetes Collaborators (2023). Global, regional, and national burden of diabetes from 1990 to 2021, with projections of prevalence to 2050: A systematic analysis for the Global Burden of Disease Study 2021. Lancet.

[13] Tinajero M.G., Malik V.S. (2021). An Update on the Epidemiology of Type 2 Diabetes: A Global Perspective. Endocrinol. Metab. Clin. N. Am..

[14] Chivese T., Hoegfeldt C.A., Werfalli M., Yuen L., Sun H., Karuranga S., Li N., Gupta A., Immanuel J., Divakar H. (2022). IDF Diabetes Atlas: The prevalence of pre-existing diabetes in pregnancy—A systematic reviewand meta-analysis of studies published during 2010–2020. Diabetes Res. Clin. Pract..

[15] Harreiter J., Roden M. (2023). Diabetes mellitus–Definition, Klassifikation, Diagnose, Screening und Prävention (Update 2023). Wien. Klin. Wochenschr..

[16] Ogurtsova K., Guariguata L., Barengo N.C., Ruiz P.L., Sacre J.W., Karuranga S., Sun H., Boyko E.J., Magliano D.J. (2022). IDF diabetes Atlas: Global estimates of undiagnosed diabetes in adults for 2021. Diabetes Res. Clin. Pract..

[17] Mestre-Font M., Busquets-Cortés C., Ramírez-Manent J.I., Tomás-Gil P., Paublini H., López-González A.A. (2024). Influence of sociodemographic variables and healthy habits on the values of type 2 diabetes risk scales. Acad. J. Health Sci..

[18] Haw J.S., Shah M., Turbow S., Egeolu M., Umpierrez G. (2021). Diabetes Complications in Racial and Ethnic Minority Populations in the USA. Curr. Diabetes Rep..

[19] Paudel G., Vandelanotte C., Dahal P.K., Biswas T., Yadav U.N., Sugishita T., Rawal L. (2022). Self-care behaviours among people with type 2 diabetes mellitus in South Asia: A systematic review and meta-analysis. J. Glob. Health.

[20] Kalan Farmanfarma K.H., Ansari-Moghaddam A., Zareban I., Adineh H.A. (2020). Prevalence of type 2 diabetes in Middle-East: Systematic review & meta-analysis. Prim. Care Diabetes.

[21] Ikechi I.S., Ejike-Odeh E.J., Ifeanyichukwu O.E., Ogbu C., Agwu U.U., Ife E. (2023). Prevalence of prediabetes among first degree relatives of type 2 diabetes individuals in Abakaliki, Ebonyi State Nigeria. Acad. J. Health Sci..

[22] Imerb N., Thonusin C., Chattipakorn N., Chattipakorn S.C. (2020). Aging, obese-insulin resistance, and bone remodeling. Mech. Ageing Dev..

[23] Tan T.E., Wong T.Y. (2023). Diabetic retinopathy: Looking forward to 2030. Front. Endocrinol..

[24] Li X., Lu L., Hou W., Huang T., Chen X., Qi J., Zhao Y., Zhu M. (2022). Epigenetics in the pathogenesis of diabetic nephropathy. Acta Biochim. Biophys. Sin..

[25] Magdaleno Marcos G., Martín Serradilla J.I., de Andrés de Llano J.I., Andrés Alberola I., Per Contreras F.E., Eirín Feijoo M., Fernández Morado C. (2023). Tendencias en la hospitalización de complicaciones neurológicas de diabetes mellitus en el área de Palencia en el periodo 1993–2017. Acad. J. Health Sci..

[26] Karagiannidis E., Moysidis D.V., Papazoglou A.S., Panteris E., Deda O., Stalikas N., Sofidis G., Kartas A., Bekiaridou A., Giannakoulas G. (2022). Prognostic significance of metabolomic biomarkers in patients with diabetes mellitus and coronary artery disease. Cardiovasc. Diabetol..

[27] Georgakis M.K., Harshfield E.L., Malik R., Franceschini N., Langenberg C., Wareham N.J., Markus H.S., Dichgans M. (2021). Diabetes Mellitus, Glycemic Traits, and Cerebrovascular Disease: A Mendelian Randomization Study. Neurology.

[28] Caruso P., Maiorino M.I., Longo M., Porcellini C., Matrone R., Digitale Selvaggio L., Gicchino M., Carbone C., Scappaticcio L., Bellastella G. (2024). Liraglutide for Lower Limb Perfusion in People With Type 2 Diabetes and Peripheral Artery Disease: The STARDUST Randomized Clinical Trial. JAMA Netw. Open.

[29] Poznyak A., Grechko A.V., Poggio P., Myasoedova V.A., Alfieri V., Orekhov A.N. (2020). The Diabetes Mellitus-Atherosclerosis Connection: The Role of Lipid and Glucose Metabolism and Chronic Inflammation. Int. J. Mol. Sci..

[30] Ma C.X., Ma X.N., Guan C.H., Li Y.D., Mauricio D., Fu S.B. (2022). Cardiovascular disease in type 2 diabetes mellitus: Progress toward personalized management. Cardiovasc. Diabetol..

[31] Aguiló Juanola M.C., López-González A.A., Tomás-Gil P., Paublini H., Tárraga-López P.J., Ramírez-Manent J.I. (2024). Influence of tobacco consumption on the values of different cardiometabolic risk scales in 418,343 spanish workers. Acad. J. Health Sci..

[32] Sastre-Alzamora T., Tomás-Gil P., Paublini H., Pallarés L., Ramírez-Manent J.I., López-González A.A. (2024). Relationship between heart age and cardiometabolic risk scales in 139634 Spanish workers. Acad. J. Health Sci..

[33] Zelig R., Samavat H., Duda P., Singer S., Feldman C., LaSalle P., Muhammad E., Touger-Decker R. (2023). Screening for diabetes risk using the diabetes risk test and point-of-care hemoglobin A1C values in adults seen in a dental clinic. Quintessence Int..

[34] Ritchie S.C., Taylor H.J., Liang Y., Manikpurage H.D., Pennells L., Foguet C., Abraham G., Gibson J.T., Jiang X., Liu Y. (2024). Integrated clinical risk prediction of type 2 diabetes with a multifactorial polygenic risk score. medRxiv.

[35] Ward E.D., Hopkins W.A., Shealy K. (2022). Evaluation of the Use of a Diabetes Risk Test to Identify Prediabetes in an Employee Wellness Screening. J. Pharm. Pract..

[36] Hu P.L., Koh Y.L., Tan N.C. (2016). The utility of diabetes risk score items as predictors of incident type 2 diabetes in Asian populations: An evidence-based review. Diabetes Res. Clin. Pract..

[37] Qian F., Mora S. (2025). Risk prediction for health outcomes in type 2 diabetes: Utility of a polysocial risk score?. Lancet Healthy Longev..

[38] Vicente-Herrero M.T., Ramírez-Iñiguez de la Torre M.V., López González A.A. (2023). Estimación del nivel de riesgo cardiometabolico relacionado con obesidad en trabajadores sanitarios españoles. Acad. J. Health Sci..

[39] Garg U.K., Mathur N., Sahlot R., Tiwari P., Sharma B., Saxena A., Jainaw R.K., Agarwal L., Gupta S., Mathur S.K. (2023). Abdominal fat depots and their association with insulin resistance in patients with type 2 diabetes. PLoS ONE.

[40] Martins F.F., Marinho T.S., Cardoso L.E.M., Barbosa-da-Silva S., Souza-Mello V., Aguila M.B., Mandarim-De-Lacerda C.A. (2022). Semaglutide (GLP-1 receptor agonist) stimulates browning on subcutaneous fat adipocytes and mitigates inflammation and endoplasmic reticulum stress in visceral fat adipocytes of obese mice. Cell Biochem. Funct..

[41] Saeed M., Ali M., Zehra T., Haider Zaidi S.A., Tariq R. (2021). Intermittent Fasting: A User-Friendly Method for Type 2 Diabetes Mellitus. Cureus.

[42] Khoramipour K., Chamari K., Hekmatikar A.A., Ziyaiyan A., Taherkhani S., Elguindy N.M., Bragazzi N.L. (2021). Adiponectin: Structure, Physiological Functions, Role in Diseases, and Effects of Nutrition. Nutrients.

[43] Liu Y., Yang Y., Xu C., Liu J., Chen J., Li G., Huang B., Pan Y., Zhang Y., Wei Q. (2023). Circular RNA circGlis3 protects against islet β-cell dysfunction and apoptosis in obesity. Nat. Commun..

[44] Dambha-Miller H., Day A.J., Strelitz J., Irving G., Griffin S.J. (2020). Behaviour change, weight loss and remission of Type 2 diabetes: A community-based prospective cohort study. Diabet. Med..

[45] Lemp J.M., Bommer C., Xie M., Michalik F., Jani A., Davies J.I., Bärnighausen T., Vollmer S., Geldsetzer P. (2023). Quasi-experimental evaluation of a nationwide diabetes prevention programme. Nature.

[46] Fernández-Figares Vicioso M.P., del Barrio Fernández J.L., López-González A.A., Ramírez-Manent J.I., Vicente Herrero M.T. (2024). Prevalencia de factores de riesgo cardiometabólico. Comparativa sector Comercio vs. Industria y variables asociadas. Acad. J. Health Sci..

[47] American Diabetes Association Professional Practice Committee (2024). 2. Diagnosis and Classification of Diabetes: Standards of Care in Diabetes-2024. Diabetes Care.

[48] Marina Arroyo M., Ramírez Gallegos I., López-González A.A., Vicente-Herrero M.T., Vallejos D., Tárraga López P.J., Ramírez Manent J.I. (2024). Equation Córdoba body fat values according to sociodemographic variables and healthy habits in 386924 Spanish workers. Acad. J. Health Sci..

[49] Gómez-Ambrosi J., Silva C., Catalán V., Rodríguez A., Galofré J.C., Escalada J., Valentí V., Rotellar F., Romero S., Ramírez B. (2012). Clinical usefulness of a new equation for estimating body fat. Diabetes Care.

[50] Yildiz T., Zuhur S., Shafi Zuhur S. (2021). Diabetes Risk Assessment and Awareness in a University Academics and Employees. Med. Bull. Sisli Etfal Hosp..

[51] Hippisley-Cox J., Coupland C. (2017). Development and validation of QDiabetes-2018 risk prediction algorithm to estimate future risk of type 2 diabetes: Cohort study. BMJ.

[52] Bird M., Cerutti S., Jiang Y., Srugo S.A., de Groh M. (2022). Implementation of the CANRISK Tool: A Qualitative Exploration Among Allied Health Professionals in Canada. Can. J. Diabetes.

[53] Latcham Z., Seereeram R., Kamalodeen A., Sanchez S., Deonarine U., Sinanan R., Mungrue K. (2010). TRAQ-D (Trinidad Risk Assessment Questionnaire for Type 2 Diabetes Mellitus): A cheap, reliable, non-invasive screening tool for diabetes. Br. J. Diabetes Vasc. Dis..

[54] Abbas M., Mall R., Errafii K., Lattab A., Ullah E., Bensmail H., Arredouani A. (2021). Simple risk score to screen for prediabetes: A cross-sectional study from the Qatar Biobank cohort. J. Diabetes Investig..

[55] Obrador de Hevia J., López-González Á.A., Ramírez-Manent J.I., Paublini Oliveira H., Tárraga López P.J., Riutord-Sbert P. (2024). Relationship between alcohol consumption and other variables with the values of different cardiovascular risk factors in 139634 Spanish workers. Acad. J. Health Sci..

[56] Chandrasekaran P., Weiskirchen R. (2024). The Role of Obesity in Type 2 Diabetes Mellitus-An Overview. Int. J. Mol. Sci..

[57] Echouffo-Tcheugui J.B., Selvin E. (2021). Prediabetes and What It Means: The Epidemiological Evidence. Annu. Rev. Public Health.

[58] Salmón-Gómez L., Catalán V., Frühbeck G., Gómez-Ambrosi J. (2023). Relevance of body composition in phenotyping the obesities. Rev. Endocr. Metab. Disord..

[59] Wu Y., Li D., Vermund S.H. (2024). Advantages and Limitations of the Body Mass Index (BMI) to Assess Adult Obesity. Int. J. Environ. Res. Public Health.

[60] Tan C., Chan K.E., Ng C.H., Tseng M., Syn N., Tang A.S.P., Chin Y.H., Lim W.H., Tan D.J.H., Chew N. (2022). DEXA Scan Body Fat Mass Distribution in Obese and Non-Obese Individuals and Risk of NAFLD-Analysis of 10,865 Individuals. J. Clin. Med..

[61] He J., Zhang B., Fan Y., Wang Y., Zhang M., Li C., Zhang L., Guo P., Zhang M. (2023). Comparison of bioelectrical body and visceral fat indices and anthropometric measures in relation to type 2 diabetes by sex among Chinese adults, a cross-sectional study. Front. Public Health.

[62] Idilman I.S., Yildiz A.E., Karaosmanoglu A.D., Ozmen M.N., Akata D., Karcaaltincaba M. (2022). Proton density fat fraction: Magnetic resonance imaging applications beyond the liver. Diagn. Interv. Radiol..

[63] Marina Arroyo M., Ramírez Gallegos I., López-González Á.A., Vicente-Herrero M.T., Vallejos D., Sastre-Alzamora T., Manent J.I.R. (2024). Usefulness of the ECORE-BF Scale to Determine Atherogenic Risk in 386,924 Spanish Workers. Nutrients.

[64] Hunt S.C., Davidson L.E., Adams T.D., Ranson L., McKinlay R.D., Simper S.C., Litwin S.E. (2021). Associations of Visceral, Subcutaneous, Epicardial, and Liver Fat with Metabolic Disorders up to 14 Years After Weight Loss Surgery. Metab. Syndr. Relat. Disord..

[65] Bensussen A., Torres-Magallanes J.A., Roces de Álvarez-Buylla E. (2023). Molecular tracking of insulin resistance and inflammation development on visceral adipose tissue. Front. Immunol..

[66] Kolb H. (2022). Obese visceral fat tissue inflammation: From protective to detrimental?. BMC Med..

[67] Yamazaki H., Tauchi S., Machann J., Haueise T., Yamamoto Y., Dohke M., Hanawa N., Kodama Y., Katanuma A., Stefan N. (2022). Fat Distribution Patterns and Future Type 2 Diabetes. Diabetes.

[68] Aragón-Herrera A., Moraña-Fernández S., Otero-Santiago M., Anido-Varela L., Campos-Toimil M., García-Seara J., Román A., Seijas J., García-Caballero L., Rodríguez J. (2023). The lipidomic and inflammatory profiles of visceral and subcutaneous adipose tissues are distinctly regulated by the SGLT2 inhibitor empagliflozin in Zucker diabetic fatty rats. Biomed. Pharmacother..

[69] Gugliucci A. (2022). Biomarkers of dysfunctional visceral fat. Adv. Clin. Chem..

[70] Cheng X., Jiang S., Pan B., Xie W., Meng J. (2023). Ectopic and visceral fat deposition in aging, obesity, and idiopathic pulmonary fibrosis: An interconnected role. Lipids Health Dis..

[71] Maskarinec G., Shvetsov Y.B., Wong M.C., Garber A., Monroe K., Ernst T.M., Buchthal S.D., Lim U., Le Marchand L., Heymsfield S.B. (2022). Subcutaneous and visceral fat assessment by DXA and MRI in older adults and children. Obesity.

[72] Tornero-Aguilera J.F., Villegas-Mora B.E., Clemente-Suárez V.J. (2022). Differences in Body Composition Analysis by DEXA, Skinfold and BIA Methods in Young Football Players. Children.

[73] Dokpuang D., Zhiyong Yang J., Nemati R., He K., Plank L.D., Murphy R., Lu J. (2023). Magnetic resonance study of visceral, subcutaneous, liver and pancreas fat changes after 12 weeks intermittent fasting in obese participants with prediabetes. Diabetes Res. Clin. Pract..

[74] Mohammad A., Ziyab A.H., Mohammad T. (2023). Anthropometric and DXA-derived measures of body composition in relation to pre-diabetes among adults. BMJ Open Diabetes Res. Care.

[75] Borga M., Ahlgren A., Romu T., Widholm P., Dahlqvist Leinhard O., West J. (2020). Reproducibility and repeatability of MRI-based body composition analysis. Magn. Reson. Med..

[76] Saponaro C., Sabatini S., Gaggini M., Carli F., Rosso C., Positano V., Armandi A., Caviglia G.P., Faletti R., Bugianesi E. (2022). Adipose tissue dysfunction and visceral fat are associated with hepatic insulin resistance and severity of NASH even in lean individuals. Liver Int..

[77] López-González A.A., Ramírez Manent J.I., Vicente-Herrero M.T., García Ruiz E., Albaladejo Blanco M., López Safont N. (2022). Prevalencia de diabesidad en población laboral española: Influencia de variables sociodemográficas y consumo de tabaco. An. Sist. Sanit. Navar..

[78] Molina-Luque R., Yañez A.M., Bennasar-Veny M., Romero-Saldaña M., Molina-Recio G., López-González Á.A. (2020). A Comparison of Equation Córdoba for Estimation of Body Fat (ECORE-BF) with Other Prediction Equations. Int. J. Environ. Res. Public Health.

[79] Okawa Y., Mitsuhashi T., Tsuda T. (2025). The Asia-Pacific Body Mass Index Classification and New-Onset Chronic Kidney Disease in Non-Diabetic Japanese Adults: A Community-Based Longitudinal Study from 1998 to 2023. Biomedicines.

